# LRRC15 Targeting in Soft-Tissue Sarcomas: Biological and Clinical Implications

**DOI:** 10.3390/cancers12030757

**Published:** 2020-03-23

**Authors:** Eytan Ben-Ami, Raul Perret, Ying Huang, Félicie Courgeon, Prafulla C. Gokhale, Audrey Laroche-Clary, Benjamin K. Eschle, Valérie Velasco, François Le Loarer, Marie-Paule Algeo, James Purcell, George D. Demetri, Antoine Italiano

**Affiliations:** 1 Sarcoma Division, Department of Medical Oncology, Dana-Farber Cancer Institute, Boston, MA 02215, USA; Eytan.BenAmi@sheba.health.gov.il (E.B.-A.); Ying_Huang2@DFCI.HARVARD.EDU (Y.H.); Prafulla_Gokhale@dfci.harvard.edu (P.C.G.); benjamink_eschle@dfci.harvard.edu (B.K.E.); george_demetri@dfci.harvard.edu (G.D.D.); 2 Department of Pathology, Institut Bergonié, 33000 Bordeaux, France; r.perret@bordeaux.unicancer.fr (R.P.); v.velasco@bordeaux.unicancer.fr (V.V.); f.le-loarer@bordeaux.unicancer.fr (F.L.L.); 3 Sarcoma Unit, Institut Bergonié, 33000 Bordeaux, France; fcourgeon@immusmol.com (F.C.); a.laroche-clary@bordeaux.unicancer.fr (A.L.-C.); 4 INSERMU1218, 33000 Bordeaux, France; 5 University of Bordeaux, 33400 Talence, France; marie-paule.algeo@u-bordeaux.fr; 6 AbbVie Biotherapeutics, Redwood City, CA 94063, USA; james.purcell@abbvie.com; 7 Ludwig Center at Harvard, Harvard Medical School, Boston, MA 02215, USA

**Keywords:** LRRC15, sarcoma, ABBV-085

## Abstract

Background: LRRC15 is a member of the LRR (leucine-rich repeat) superfamily present on tumor-associated fibroblasts (CAFs) and stromal cells. The expression of LRRC15 is upregulated by the pro-inflammatory cytokine TGFβ. ABBV-085 is a monomethyl auristatin E (MMAE)-containing antibody-drug conjugate (ADC) designed to target LRRC15, and which has shown significant anti-tumor activity in several tumor models. This is the first focused examination of LRRC15 expression and ABBV-085 activity in soft-tissue sarcomas (STS). Methods: We analyzed the LRRC15 expression profile by immunohistochemistry in 711 STS cases, covering a broad spectrum of STS histologies and sub-classifications. In vivo experiments were carried out by using LRRC15-positive and LRRC15-negative patient-derived xenograft (PDX) models of STS. Results: In contrast to patterns observed in epithelial tumors, LRRC15 was expressed not only by stromal cells but also by cancer cells in multiple subsets of STS with significant variations noted between histological subtypes. Overexpression of LRRC15 is positively correlated with grade and independently associated with adverse outcome. ABBV-085 has robust preclinical efficacy against LRRC15 positive STS patient-derived xenograft (PDX) models. Conclusion: We provide the first preclinical evidence that LRRC15 targeting with an antibody-drug conjugate is a promising strategy in LRRC15-positive STS. ABBV-085 is being evaluated in an ongoing clinical trial in STS and other malignancies.

## 1. Introduction

Soft-tissue sarcomas (STS) represent approximately 1% of cancers in adults and 15% in children, and are among the most devastating of human malignancies. Indeed, despite adequate loco-regional treatment, up to 40% of patients will develop metastatic disease. Doxorubicin-based chemotherapy has been the mainstay of treatment in the advanced setting for many years; however, the median overall survival of patients is only 12–18 months. This poor outcome is in part explained by the limited sensitivity of STS to cytotoxic drugs [1]. Despite several attempts to identify new therapeutic strategies, there has been no significant improvement in the overall survival (OS) [2]. Therefore, there is an urgent need to assess new drugs with the potential to improve patient outcomes.

STS are characterized by high stromal content which may play a crucial role in resistance to chemotherapy. Several lines of evidence indicate that extracellular matrix (ECM) proteins produced by cancer-associated fibroblasts (CAF) impair chemotherapy delivery and create an immunosuppressive context that promotes progression of several solid tumors [3,4,5,6]. Therefore, the addition of anti-stroma therapies is expected to increase efficacy of chemotherapy and improve patient outcome. 

LRRC15, a 581 amino acid type I membrane protein with no obvious intracellular signaling domains, has recently been reported as a marker of cancer-associated fibroblasts [7]. This protein has been found to be highly expressed on CAFs within the tumor stroma of many tumor types, as well as directly on cancer cells in tumors of mesenchymal origin such as sarcomas [7]. Moreover, ABBV-085, a monomethyl auristatin E (MMAE)-containing antibody–drug conjugate (ADC) designed to target against LRRC15, has shown significant anti-tumor activity in several LRRC15 stromal-positive or cancer-positive models [7]. 

We report here the first detailed evaluation of the expression and prognostic impact of LRRC15 in STS as well as the preclinical activity of ABBV-085 in patient-derived xenograft models of STS. 

## 2. Materials and Methods

### 2.1. Study Reagents

ABBV-085 (therapeutic anti-LRRC15 ADC), Isotype-ADC, LRRC15 mAb-1 (anti-LRRC15 antibody for flow cytometry), Isotype antibody IgG1 (antibody control for cytometry), and LRRC15 mAb-5 (anti-LRRC15 antibody for IHC and Western Blot (WR) were supplied by AbbVie (Redwood City, CA, USA) [7].

### 2.2. Patients and Samples

We analyzed the expression of LRCC15 by immunohistochemistry in three independent cohorts of primary STS: (i) one cohort comprising 410 patients with STS harboring complex genomic aberrancies (tissue microarray), (ii) one cohort of 87 patients with leiomyosarcoma (LMS) (tissue microarray), (iii) one cohort including 214 patients with different histotypes of STS (whole slide). All the cases were reviewed by a pathologist expert in the field of soft-tissue tumors. 

### 2.3. Immunohistochemistry (IHC)

IHC on cryosection xenograft tissues fixed with cold acetone was performed using Dako REAL^TM^ EnVision^TM^ Detection System, Peroxidase/DAB+, Rabbit/Mouse (K5007). Macrophages were stained with rat anti-mouse F4/80 (BD bioscience, BD 552958, 1/100 overnight). A fixative rabbit monoclonal [R18-2] anti-rat IgG Fc (ab125900, 1/2000, 1 h) was used before adding secondary antibody anti-rabbit IgG coupled with HRP (Horse Raddish Peroxydase). DAB (3,3′-Diaminobenzidine) was used to detect bound primary antibodies. Dako Hematoxylin was used to counter stain nuclei. Images were acquired on a Panoramic 250 Flash III Digital Slide Scanner (3DHISTECH^TM^). IHC for LRRC15 expression on human tumor samples (LRRC15 mAb-5, 1 µ/mL, 40 min) was performed with Ultraview/Ventana system after a CC1 light (cell conditioning 1 light) process (96 °C, 36 min). LRRC15 expression was assessed blindly by a soft tissue pathologist (P.R.) using the H-score. This score is obtained by multiplying the percentage of total positive cells by the predominant staining intensity (scored from 0 for “no signal” to 3 for “strong signal”), 300 values are therefore possible. The total tumor area and the stromal compartment (fibroblasts, endothelial cells, inflammatory cells, and extracellular matrix) within this surface was evaluated in all cases.

### 2.4. In Vivo Studies

Patient derived xenografts: independent in vivo experiments were performed in two different animal facilities (Dana Farber Cancer Institute and Institut Bergonié) to verify the reproducibility of the efficacy results. Experiments were performed on NOD scid gamma (NSG™) mice, following approval of the Institutional Animal Care and Use Committee (IACUC) in an AAALAC-accredited vivarium and at the University of Bordeaux, respectively. The activity of ABBV-085 was tested in six sarcoma patient-derived xenograft models including liposarcoma (DDLPS), leiomyosarcoma and undifferentiated sarcomas (UPS) with varying LRRC15 expression. Surgical samples were obtained from patients following their consent according to the Institutional Review Board-approved protocol of Dana Farber Cancer Institute (IRB-23KN54) and Institut Bergonié (IRB-2018C4), respectively. Tumor fragments dipped in Matrigel (Fisher Scientific, NH) were maintained subcutaneously by serially passaging in NSG mice. For efficacy study, tumors were allowed to establish to 200 ± 50 mm^3^ in size before randomization into various treatment groups with 7–9 mice per group. Isotype-control, isotype-MMAE, and ABBV-085, diluted in PBS were administered at 6 mg/kg once every 4 days intraperitoneally for a total of six injections. Tumor volumes were determined from caliper measurements by using the formula V = (length × width^2^)/2. Tumor sizes and body weight were measured twice weekly. Mice were treated over 24 days, followed by measuring for re-growth of tumors.

### 2.5. Statistical Analysis

The statistical analysis of baseline demographics and clinical outcomes is based on all data available up to the cut-off date of 31 December 2017. Survival rates were estimated with the Kaplan–Meier method. Differences between groups were evaluated by the χ^2^ test or Fisher’s exact tests for categorical variables and *t*-tests for continuous variables. Univariate and multivariate analyses with a Cox regression model were used to identify prognostic factors. Variables tested in univariate analysis included age; gender; tumor site (limb versus other); tumor location (deep versus superficial); histological grade; and tumor size. Variables associated with PFS at *p* < 0.05 in the univariate analysis were included in the multivariate regression. Analyses were performed using SPSS 19.0 statistical software (IPSS Inc., Chicago, IL, USA). All statistical tests were two-sided, and *p* < 0.05 indicated statistical significance.

## 3. Results

### 3.1. LRRC15 Is Highly Expressed in Several Histological Sarcomas Subtypes

**Figure 1:**
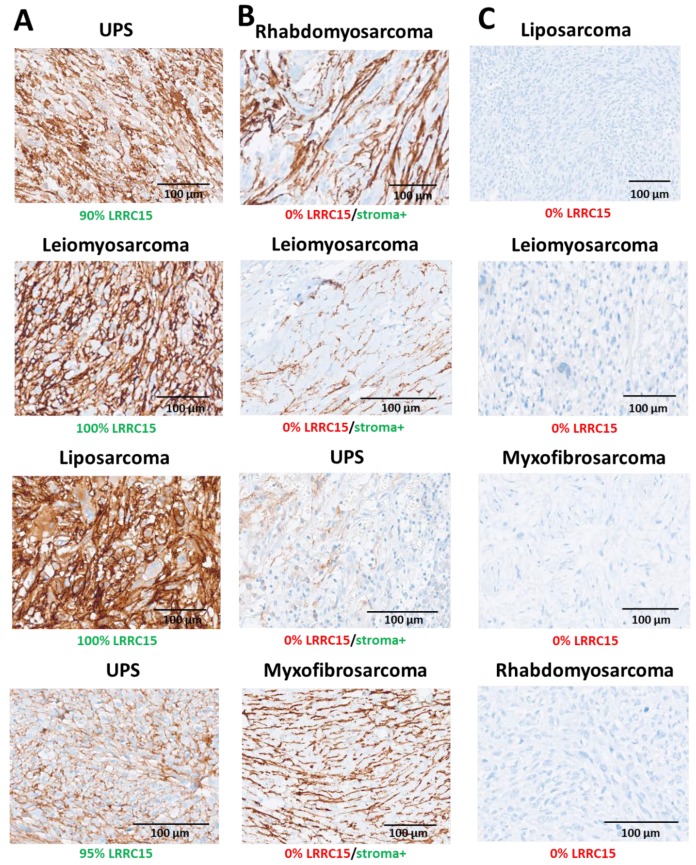
LRRC15 staining obtained by immunohistochemistry for different histological subtypes of soft-tissue sarcomas (STS) with different level of expression in cancer cells and stroma. (**A**) Examples of STS with a significant expression in cancer cells. (**B**) Examples of STS without expression of LRRC15 in cancer cells but with an expression in the surrounding stroma. Staining was predominantly seen in spindle cells (fibroblasts) and in the extracellular matrix. (**C**) Examples of STS without expression of LRRC15.

We analyzed LRRC15 protein expression by IHC in 711 cases of STS, including gastrointestinal stromal tumors (GIST). The specificity of the antibody used was already extensively tested on several positive/negative tumor cell lines, and assessed by orthogonal methods (Western blotting/flow cytometry) and using CRISPR technology [7]. In contrast to the patterns observed in epithelial tumors, LRRC15 was expressed not only by normal stromal (predominantly fibroblasts) cells but also by cancer cells. Results are described in Table 1 and illustrated in Figure 1. The proportion of LRRC15-positive cases differed significantly according to histological subtypes with staining observed in 51%, 47%, and 36% of UPS, dedifferentiated liposarcomas and leiomyosarcomas, respectively (*p* = 0.003). UPS was the histological subtype with the highest proportion of strong expression (25%) followed by leiomyosarcomas (19%) and dedifferentiated liposarcomas (14%), *p* = 0.06. The proportion of LRRC15-positive cases among myxofibrosarcomas was significantly lower in comparison to other histological subtypes such as UPS (19%, *p* < 0.0001) (Table 1). A total of 424 cases have available histological grade data. LRRC15 expression was also correlated with histological grade. Additionally, 21% of grade 3 tumors are characterized by a high expression of LRRC15 versus only 10% of grade 2 tumors and 6% of grade 1 tumors, *p* < 0.001 (Supplementary Table S1).

### 3.2. High Expression of LRRC15 by Sarcomas Cells Is Associated with Adverse Outcome

**Figure 2:**
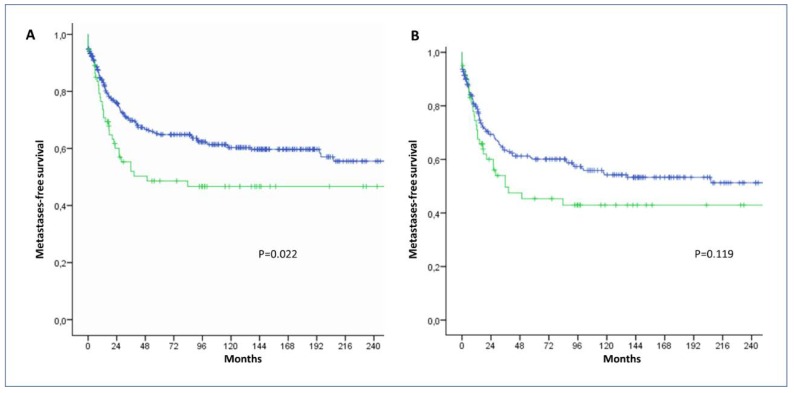
Kaplan-Meier curves of metastases-free survival according LRRC15 expression. (**A**) Kaplan–Meier curves of metastases-free survival according to LRRC15 expression score in a cohort of 425 patients with soft-tissue sarcoma (green line: LRRC15 H score > 160, blue line: LRRC15 H score ≤ 160). (**B**) Kaplan–Meier curves of metastases-free survival according to LRRC15 expression (green line: LRRC15 H score > 160, blue line: LRRC15 H score ≤ 160) in patients with grade 3 STS.

By analyzing the LRRC15 expression profile of a series of 425 STS with available clinical data (Table S1), we found that high LRRC15 expression was significantly associated with worse metastases-free survival in multivariate analysis (Figure 2 and Table 2). As described in Figure 2, LRRC15 sarcoma cell expression provided extra information to further refine the prognosis of patients besides histological grade which is considered the most significant predictor of outcome. 

### 3.3. ABBV-085 Has Anti-Tumor Activity in LRRC15-Positive Sarcoma Models

**Figure 3:**
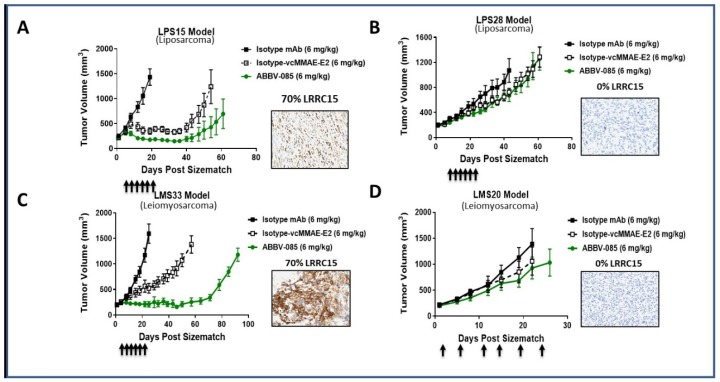
In vivo anti-tumor activity of ABBV-085 monotherapy in STS. (**A**,**B**) Tumor growth curves for liposarcoma models LPS15 (A: LRRC15 positive) and LPS28 (B: LRRC15 negative) treated with ABBV-085. (**B**) Tumor growth curves for leiomyosarcoma models LMS33 (**C**: LRRC15 cancer positive) and LMS20 (**D**: LRRC15 negative) treated with ABBV-085. A total of 7–9 mice/group of treatment. For each model, a figure indicating the level LRRC15 expression in the parental tumor from which the patient-derived xenograft (PDX) was derived is represented.

Given the high significant expression of LRRC15 by sarcoma cells and its prognostic impact, we assessed the efficacy of ABBV-085, the first-in-class antibody–drug conjugate (ADC) directed against LRRC15, in several patient-derived xenograft models of UPS, LMS, and DDLPS. As shown, in Figure 3, ABBV-085 demonstrated significant antitumor activity in multiple LRRC15 STS-positive models. For the UPS (Supplementary Figure S1) and LMS models, treatment with ABBV-085 resulted in significant tumor growth inhibition and tumor growth delay compared with isotype control and cytotoxic control groups. Interestingly, in the UPS models (where the highest LRRC15 expressions were observed), profound tumor regressions were observed, with complete tumor regression and no tumor regrowth during extended monitoring period, including one model (JR588) which was refractory to STS current therapies (doxorubicin, ifosfamide, gemcitabine) (Supplementary Figure S1). Tumor growth inhibition was associated with a dramatic reduction in the number of LRRC15-expressing tumor cells (Supplementary Figure S3). In models with no LRRC15 expression (0% positivity), minimal tumor growth inhibition and no significant differences were observed between any of the treatment arms in these groups. 

## 4. Discussion

We report here the first large study assessing LRRC15 expression in mesenchymal malignancies (soft-tissue sarcomas) and its potential therapeutic application in this indication. LRRC15 has been shown to be highly expressed on CAFs within the tumor stroma of many epithelial tumors. By analyzing a small cohort of 39 STS, we have also observed that LRRC15 can also be directly expressed on cancer cells from mesenchymal tumors [7]. Consistent with these first results, we found LRRC15 to be expressed by cancer cells in up to 36% of STS with significant differences across histological subtypes (Table 1, Figure 1). For instance, 50% of UPS expressed LRRC15 versus 19% only for myxofibrosarcoma. Historically, MFS was considered a subset of UPS (“myxoid malignant fibrous histiocytoma”), but more recently, MFS and UPS have been classified as distinct tumor types based on their different clinico-pathologic characteristics [8]. MFS has prominent myxoid stroma and is often lower grade and prone to local relapse, while UPS is generally higher grade, more cellular, and prone to distant metastasis and worse outcome. The molecular determinants of such differences in expression between histological subtypes remains to be elucidated. However, this may be related to differential expression of transforming growth factor β (TGF-β) between histological subtypes. Indeed, LRRC15 expression is tightly regulated by TGF-β [7]. TGF-β is a multifunctional growth factor that variably affects proliferation, differentiation, and extracellular matrix formation, resulting in an immunosuppressive microenvironment. Although there is no large dataset describing the expression of this protein in STS, recent studies have shown a strong expression in UPS whereas expression is reported to be low in MFS [9,10]. Interestingly, we also found a differential expression of LRRC15 according to grade with a higher expression in grade 3 versus grade 1 or 2 tumors. Strikingly, high expression of LRRC15 has an independent prognostic value. Whether this adverse prognostic impact is linked to activation of cell-autonomous growth pathways and/or modification of the tumor microenvironment will require additional investigations. 

We have demonstrated ABBV-085 monotherapy activity in all LRRC15 positive patient-derived xenograft (PDX) models of STS, even in highly chemo-refractive indications such as UPS (PDX; Supplementary Figure S1) with poor treatment options and survival [1]. Interestingly, we found in several models that tumors do not regrow even several weeks after the last administration of ABBV-085. Despite the obvious efficacy of ABBV-085 to reduce tumor growth in tumors with high expression of LRRC15, we could observe, in some STS PDX models, a similar activity with the non-binding control (hIgG-MMAE). This observation could be explained by the cross-reactivity of ABBV-085 in mice compared with the non-mouse cross reactive isotype-MMAE (resulting in a significantly shorter half-life of ABBV-085 compared with isotype = MMAE, ≈2–3 days vs. 10+days, respectively). However, previous studies suggest that tumor-associated macrophages (TAMs) are involved in the antitumor activity of ADC through FcγR interaction [11]. In order to test this hypothesis, we assessed the level of macrophage infiltration in the UPS JR588 PDX model by using IHC and found a strong infiltration (Supplementary Figure S2). This suggests that as reported for other ADCs, TAM may contribute to the anti-tumor activity of ABBV-085. We have recently reported the importance of TAM infiltration in STS, its prognostic impact as well as its role in primary resistance to PD1 inhibition [12,13]. ABBV-085 has been shown to increase immune infiltration in preclinical models [7]. Further studies should focus on the role of TME in the anti-tumor activity of LRRC15 targeting in sarcomas as well as the potential therapeutic values of strategies combining ABBV-085 with PD-1 or PD-L1 inhibition particularly in UPS, which appears to be one of the STS subtypes that is the most sensitive to such immune checkpoint inhibitors [14].

Recently, results of a phase 1, first-in-human, clinical trial of ABBV-085 monotherapy in patients (patients) with advanced solid tumors (NCT02565758) were presented [15]. A total of 78 patients were enrolled in this study, of which 27 patients with advanced sarcomas UPS (*n* = 10), osteosarcoma (*n* = 10), other sarcomas (*n* = 7) were treated in the dose-escalation (*n* = 8) or dose-expansion cohorts (*n* = 19). Overall, ABBV-085 was well tolerated, with grade ≥3 treatment-emergent adverse events reported in 56 (71.8%) patients (most commonly anemia (14.1%)), and dose-limiting toxicities of anemia (*n* = 1), hypertriglyceridemia (*n* = 1), and ileus and nausea (*n* = 2). Of the 27 sarcoma patients, four (14.8%) had confirmed partial response and eight (29.6%) had stable disease, with a median duration of response (confirmed responders) of 7.6 months. 

In summary, LRRC15 represents a promising new therapeutic target in STS based on these data. ABBV-085 is a first-in-class stromal targeting ADC which was well-tolerated in a phase 1 study in patients with advanced sarcomas, with durable partial responses observed in these patients. Given its efficacy and safety profile, further combinations of ABBV-085 with immune checkpoint inhibitors are very intriguing, particularly in UPS, which appears to be one of the STS subtypes that is the most sensitive to ABBV-085 and PD-1/PD-L1 checkpoint inhibitors. 

## Figures and Tables

**Table 1:** LRRC15 expression in soft-tissue sarcoma.

Cancer Type	*n*	Cancer Cell Positivity: LRRC15 IHC Score (*n* = 711)				Cancer Cell and/or Stroma Positivity: LRRC15 IHC Score (*n* = 711)		
		0% cells	<2+ and/or ≤ 50% cells	≥2+ and >50% cells	Strong Positivity (%)	0+	≥1+	Median Score
Soft-Tissue Sarcomas	711	452	143	116		299	412	
UPS	142	70	36	36	25	46	96	175 CI: 120–225
Leiomyosarcoma	288	184	48	56	19	124	164	270 CI: 195–290
Liposarcoma	72	38	24	10	14	29	43	60 CI: 45–80
Myxofibrosarcoma	68	55	8	5	7	36	32	110 CI: 80–140
GIST	76	68	8	0	0	48	28	80 CI: 70–100
Rhabdomyosarcoma	17	14	2	1	6	8	9	20 CI: 5–35
Other	48	23	17	8	17	8	40	150 CI: 110–175

CI: confidence interval, UPS: undifferentiated pleomorphic sarcomas, GIST: gastrointestinal stroma tumor.

**Table 2:** Multivariate analysis for metastasis-free survival (*n* = 425).

	HR	*p*
Tumor size < 5 cm	0.49	0.0034
Histological subtype		
Leiomyosarcoma	1.74	0.001
FNCLCC grade		
Grade 1	0.26	0.039
Grade 2	0.67	
LRRC15 IHC score		
>160 (median score)	0.66	0.043

## Supplementary Materials

The following are available online at https://www.mdpi.com/2072-6694/12/3/757/s1, Figure S1: Preclinical activity of ABBV-085 in LRRC15-positive UPS patient derived xenograft models, Figure S2: Immunohistostaining of macrophages (F4/80) on PDX JR588 paraffin slices of treated mouse by (A) Vehicle, (B) Isotype ADC, (C) ABBV-085 antibody, Figure S3: LRRC15 expression on PDX JR588 paraffin slices of treated mouse by (A) ABBV-085, (B) Vehicle (C) Isotype. Table S1: LRRC15 expression according to grade.

## References

[1] Savina M., Le Cesne A., Blay J.Y., Ray-Coquard I., Mir O., Toulmonde M., Cousin S., Terrier P., Ranchere-Vince D., Meeus P. (2017). Patterns of care and outcomes of patients with METAstatic soft tissue SARComa in a real-life setting: The METASARC observational study. BMC Med..

[2] Italiano A., Mathoulin-Pelissier S., Cesne A.L., Terrier P., Bonvalot S., Collin F., Michels J.J., Blay J.Y., Coindre J.M., Bui B. (2011). Trends in survival for patients with metastatic soft-tissue sarcoma. Cancer.

[3] Turley S.J., Cremasco V., Astarita J.L. (2015). Immunological hallmarks of stromal cells in the tumour microenvironment. Nat. Rev. Immunol..

[4] Feig C., Gopinathan A., Neesse A., Chan D.S., Cook N., Tuveson D.A. (2012). The pancreas cancer microenvironment. Clin. Cancer Res..

[5] Olive K.P., Jacobetz M.A., Davidson C.J., Gopinathan A., McIntyre D., Honess D., Madhu B., Allard D., Feig C., Chang A. (2009). Inhibition of Hedgehog signaling enhances delivery of chemotherapy in a mouse model of pancreatic cancer. Science.

[6] Berchtold S., Grunwald B., Kruger A., Reithmeier A., Hahl T., Cheng T., Born D., Erkan M., Kleeff J., Esposito I. (2015). Collagen type V promotes the malignant phenotype of pancreatic ductal adenocarcinoma. Cancer Lett..

[7] Purcell J.W., Tanlimco S.G., Hickson J., Fox M., Sho M., Durkin L., Uziel T., Powers R., Foster K., McGonigal T. (2018). LRRC15 Is a Novel Mesenchymal Protein and Stromal Target for Antibody-Drug Conjugates. Cancer Res..

[8] Fletcher C.D.M., Bridge J.A., Hogendoorn P.C.W., Mertens F. (2013). Classification of Tumours of Soft Tissue and Bone Classification of Tumours.

[9] Yamamoto T., Akisue T., Marui T., Fujita I., Matsumoto K., Hitora T., Kawamoto T., Nagira K., Nakatani T., Kurosaka M. (2004). Expression of transforming growth factor beta isoforms and their receptors in malignant fibrous histiocytoma of soft tissues. Clin. Cancer Res..

[10] De Vita A., Recine F., Mercatali L., Miserocchi G., Liverani C., Spadazzi C., Casadei R., Bongiovanni A., Pieri F., Riva N. (2017). Myxofibrosarcoma primary cultures: Molecular and pharmacological profile. Ther. Adv. Med. Oncol..

[11] Li F., Ulrich M., Jonas M., Stone I.J., Linares G., Zhang X., Westendorf L., Benjamin D.R., Law C.L. (2017). Tumor-Associated Macrophages Can Contribute to Antitumor Activity through FcγR-Mediated Processing of Antibody-Drug Conjugates. Mol. Cancer Ther..

[12] Toulmonde M., Adam J., Bessede A., Ranchère-Vince D., Velasco V., Brouste V., Blay J.-Y., Mir O., Italiano A. (2016). Integrative assessment of expression and prognostic value of PDL1, IDO, and kynurenine in 371 primary soft tissue sarcomas with genomic complexity. J. Clin. Oncol..

[13] Toulmonde M., Penel N., Adam J., Chevreau C., Blay J.Y., Le Cesne A., Bompas E., Piperno-Neumann S., Cousin S., Grellety T. (2018). Use of PD-1 Targeting, Macrophage Infiltration, and IDO Pathway Activation in Sarcomas: A Phase 2 Clinical Trial. JAMA Oncol..

[14] Tawbi H.A., Burgess M., Bolejack V., Van Tine B.A., Schuetze S.M., Hu J., D’Angelo S., Attia S., Riedel R.F., Priebat D.A. (2017). Pembrolizumab in advanced soft-tissue sarcoma and bone sarcoma (SARC028): A multicentre, two-cohort, single-arm, open-label, phase 2 trial. Lancet Oncol..

[15] Demetri G.D., Luke J.J., Hollebecque A., Powderly J.D., Spira A.I., Subbiah V., Purcell J., Lai D.W., Yue H., Myzak M. (2019). First-in-human phase 1 study of ABBV-085, an antibody-drug conjugate (ADC) targeting LRRC15, in sarcomas and other advanced solid tumors. J. Clin. Oncol..

