# Supplementary material for: LRRC15 Targeting in Soft-Tissue Sarcomas: Biological and Clinical Implications
Source: Cancers (Basel). 2020 Mar 23;12(3):757. doi: 10.3390/cancers12030757 (PMC7140027; doi:10.3390/cancers12030757)
Supplement: Supplementary file 1 [file cancers-12-00757-s001.pdf]

cancers

MDPI

Article

LRRC15 Targeting in Soft-Tissue Sarcomas: Biological and Clinical Implications

Eytan Ben-Ami, Raul Perret, Ying Huang, Félicie Courgeon, Prafulla Gokhale, Audrey Laroche-Clary, Benjamin Eschle, Valérie Velasco, François Le Loarer, Marie-Paule Algeo, James Purcell, George Demetri and Antoine Italiano

Supplementary Materials

Supplementary Table S1. LRRC15 expression according to grade.

Tumour characteristic		LRRC15 expression Low *	LRRC15 expression High *	Total
Grade	**1**	17	1	18
	2	112	13	125
	3	221	60	281
Total		350	74	424

* Low LRRC15 expression (H score ≤ 160); High LRRC15 expression (H score > 160).

(**A**) (**B**)

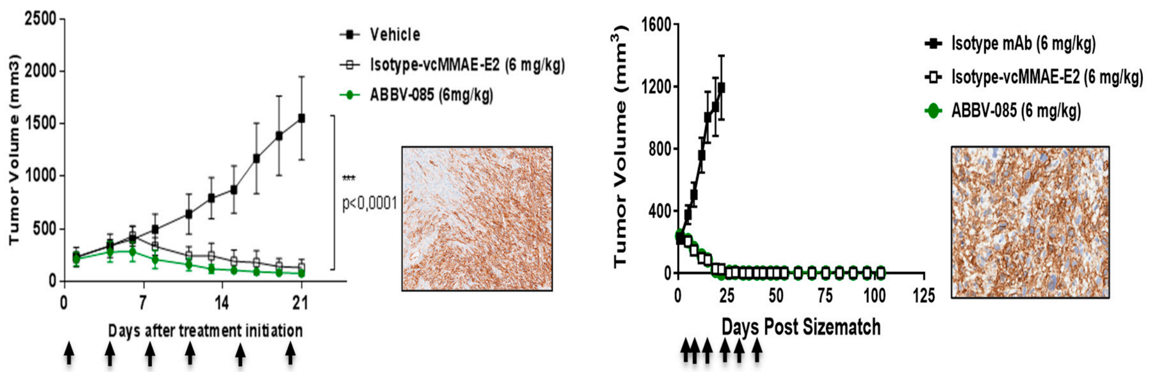

Supplementary Figure S1. Preclinical activity of ABBV-085 in LRRC15-positive UPS patient derived xenograft models (**A**: JR588 and **B**: UPS7).

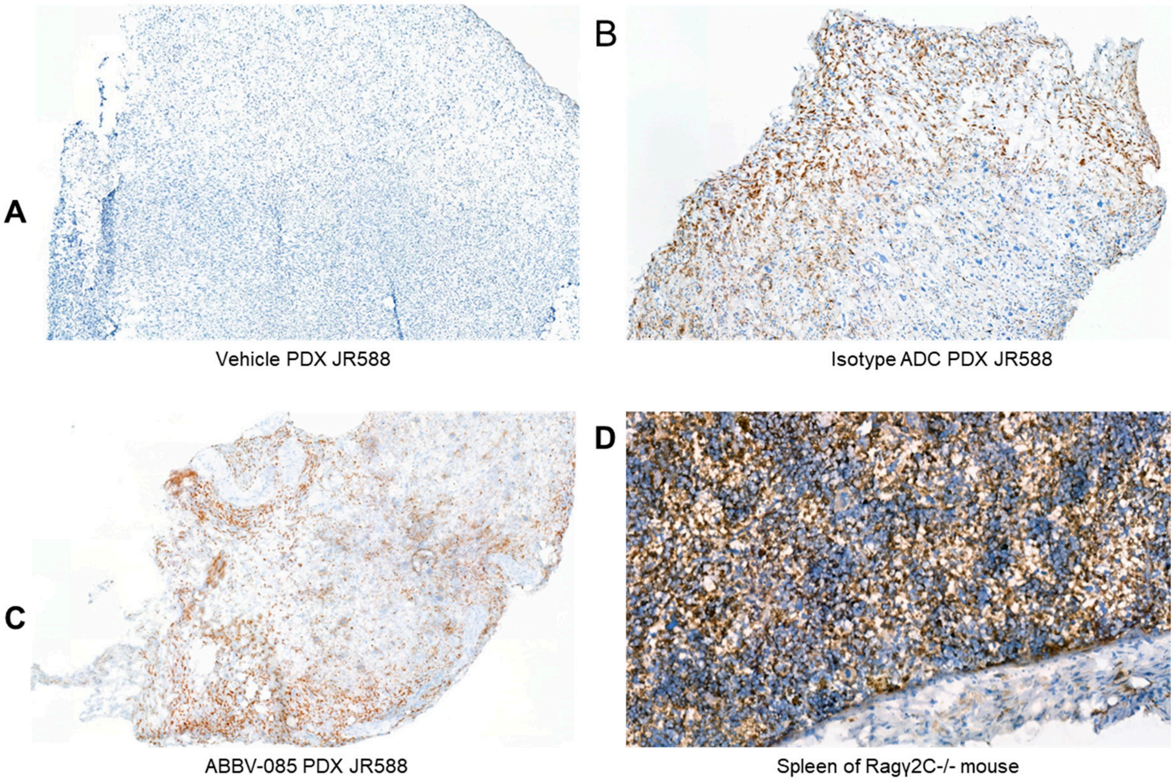

Supplementary Figure S2. Immunohistostaining of macrophages (F4/80) on PDX JR588 paraffin slices of treated mouse by (**A**) Vehicle, (**B**) Isotype ADC, (**C**) ABBV-085 antibody. (**D**) Control of F4/80 immunostaining on a Ragγ2C-/- spleen paraffin slice.

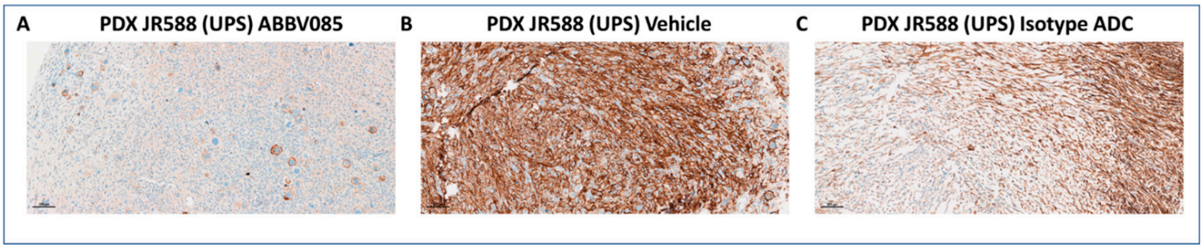

Supplementary Figure S3. LRRC15 expression on PDX JR588 paraffin slices of treated mouse by (**A**) ABBV-085, (**B**) Vehicle (**C**) Isotype.

© 2020 by the authors. Licensee MDPI, Basel, Switzerland. This article is an open access article distributed under the terms and conditions of the Creative Commons Attribution (CC BY) license (http://creativecommons.org/licenses/by/4.0/).
